# Book Reviews

**Published:** 1898-11

**Authors:** 


### BOOK REVIEWS, PAMPHLETS, EXCHANGES.

### BOOK REVIEWS.

[Contributions solicited for review.]

A TEXT-BOOK OF PRACTICAL THERAPEUTICS: With especial Reference to the Application of Remedial Measures to Disease and their Employment upon a Rational Basis. By Hobart Amory Hare, M.D., Professor of Therapeutics and Materia Medica in the Jefferson Medical College of Philadelphia. With special chapters by Drs. G. E. de Schweinitz, Edward Martin and Barton C. Hirst. New (seventh) edition. In one octavo volume of 770 pages, illustrated. Cloth, $3.75; leather, $4.50, net. Lea Brothers & Co., Philadelphia and New York.

The fact of the text-book having reached its seventh edition in so short a time after its first appearance is an unquestioned evidence of its popularity, and this popularity is the very best evidence of its worth.

The writer was for nineteen years Professor of Materia Medica and Therapeutics in a reputable college, and began the use of this excellent work with its first edition—as a text-book—and were he a professor for the next twenty years to come, with the necessary revisions and additions of the author continued, which have been up to date so faithfully done, he would still use it.

The teacher and student owe Professor Hare a debt of gratitude for his efforts in their behalf in the field of materia medica and therapeutics, especially for the lucid and simple manner in which he applies the therapeutics to the remedies treated of. With each remedy he gives the student an object-lesson, in the disease for which it is applicable, the mode and dose of administration, and usually a formula, either simple or compound, in which it may be used. This is of great benefit and value to the advanced student and young practitioner, and the recognition of this fact by them is one cause of its great popularity. As a teacher, I cannot too highly recommend this book to students, and as an active practitioner of over forty years' experience, I can say the same to my professional brothers—the busy doctor. The addition in the seventh edition of the colabors of such learned scholars as Drs. G. E. de Schweinitz, Edward Martin and Barton C. Hirst has enhanced the value of the book. G. G. R.

---

A MANUAL OF OTOLOGY. By Gorham. Bacon, A.M., M.D., Professor of Otology in Cornell University Medical College, New York. With an Introductory Chapter by Clarence J. Blake, M.D., Professor of Otology in the Harvard Medical School, Boston. In one handsome 12mo volume of 400 pages, with 109 engravings and 1 colored plate. Cloth, $2.00, net. Lea Brothers & Co., Publishers, Philadelphia and New York.

This Manual of Otology is before us. As a manual it will no doubt meet the wants of a great many students, and then again, what is a great desideratum to many of this class, it is not an expensive book. The great fault to us, however, is the fact that it is even too compact for a manual. Such illustrations as are given, it is true, are most excellent, but they have all been taken from works of a more extensive character, and even most of these represent the various instruments which are used and are good advertisements for the instrument-makers. The work has an introduction by Dr. Clarence J. Blake, of Boston, Mass., one of the foremost Otologists in this country, but just what Dr. Bacon expected to gain for his work by this three and a quarter page introduction, we fail to see. We hold that there are too many text-books and manuals published these days, and in many cases the only reason for such is because its author wishes to see his name thus associated in print. This manual is an excellent compilation of the present views as held by the best workers in Otology, and occasionally the author has given some of his own clinical experience. Dr. Bacon stands high as an Otologist, and no one has done better work than he has at the New York Eye and Ear Infirmary, but we do not think that he has added much to his fame by the publication of his little book. It is a safe guide, and for this reason can be recommended. The teachings are exceedingly practical.

PRACTICAL DIAGNOSIS. The Use of Symptoms in the Diagnosis of Disease. By Hobart Amory Hare, M.D., Professor of Therapeutics and Materia Medica in the Jefferson Medical College of Philadelphia. Third edition, enlarged and thoroughly revised. In one octavo volume of 615 pages, with 204 engravings and 13 full-page colored plates. Cloth, $4.75, net. Lea Brothers & Co., Publishers, Philadelphia and New York.

Very little more can be said for this work than the fact that this is its third edition issued in the space of two years. This speaks for itself, and shows in what value it is held by the medical profession. Dr. Hare is an exceedingly practical man, besides understanding the subject thoroughly from a scientific standpoint, and with these two qualities combined, he has produced a work on diagnosis which is unusually attractive and instructive in character. It is a companion piece to his work on "Practical Therapeutics," which is also reviewed in this issue, and with these two books in his library, a physician possesses a condensed encyclopedia of all those points which will be of most use to him in the practice of medicine. This work has been received with great favor in Great Britain, and it must be most gratifying to its author to know that his English cousins appreciate its value. In the last edition a goodly number of photographic plates have been added, showing some of the clinical cases in Jefferson College Hospital under the author's care, and this has made a new and attractive feature. But above all, Dr. Hare's language is so clear and put with such striking force, that one who reads must understand the subject.

---

A TEXT-BOOK OF MATERIA MEDICA, THERAPEUTICS AND PHARMACOLOGY. By George Frank Butler, Ph.G., M.D., Professor of Materia Medica and Clinical Medicine in the College of Physicians and Surgeons, Chicago. Published by W. B. Saunders, Philadelphia. Price, Cloth, $4.00; sheep, $5.00.

When we consider the fact that there are a large number of excellent Text-Books on Materia Medica, written by men who are eminently qualified, and the fact that the author was compelled to issue this the second edition of his work within two years after its first publication, then we are compelled to recognize the fact that

Dr. Butler has given to the profession a work which has been duly appreciated. There are, of course, works which are more exhaustive, but for the student we must commend this work for its conciseness, practicality and for the thorough up-to-date manner in which the subjects are treated. The arrangement of the book is eminently practical.

The author treats first of *Pharmacology and General Therapeutics*, followed by *Pharmaceutical Preparations*, which are taken up in detail.

Then comes Class I.—Disease Medicines.

Class II.—Symptom Medicines, and finally *Topical Remedies*, followed with a very practical discourse upon *Prescriptions*, a subject too often neglected by most works on Materia Medica.

---

R. L. POLK & CO.'S MEDICAL AND SURGICAL REGISTER OF THE UNITED STATES AND CANADA. Fifth Revised Edition. R. L. Polk & Co. Detroit & Chicago.

This edition of a well-known work is now out, and is extended in its scope to include a registry of the physicians of the Dominion of Canada. Everything pertaining to location, school and graduation of all kinds of doctors appears in its pages. All State medical laws, all societies, army and navy medical men, in fact almost everything suitable for a directory of and for physicians is to be found in this book. Errors, such as refers to a dead medical journal here, are to be found. Where the pubishers have been notified of error about a doctor they have sent out correction slips to be pasted over the original. We consider the book indispensable.

